# Implicit motor learning in children with autism spectrum disorder: current approaches and future directions

**DOI:** 10.3389/fpsyt.2024.1253199

**Published:** 2024-04-05

**Authors:** Weiqi Zheng

**Affiliations:** School of Psychology, Beijing Sport University, Beijing, China

**Keywords:** implicit motor learning, analogy learning, focus of attention, autism spectrum disorder, children

## Abstract

Motor dysfunction is increasingly being viewed as a core characteristic of autism spectrum disorder (ASD) in children. In particular, children with ASD have difficulty in learning new motor skills and there is a need to develop effective methods to improve this. Previous research has found that children with ASD may retain the ability to implicitly learn motor skills in comparison to their explicit learning of motor skills, which is typically impaired. This literature mini review focuses on summarizing the study of implicit learning in the acquisition of motor skills in children with ASD. First, we briefly introduce several common implicit learning methods in children’s motor skill learning. Second, we focus on the role of two important implicit learning approaches in motor skill learning, namely, an external focus of attention and analogy learning. Finally, based on our review of the existing studies, we present an outlook for future research and the areas that need to be improved in the practical teaching of implicit learning in the acquisition of motor skills in children with ASD.

## 1 Introduction

Autism spectrum disorder (ASD) is a pervasive neurological developmental disorder that often diagnosed in early childhood and is characterized by difficulties with social interaction and communication, inflexible behavior and thinking, repetitive and restrictive behavior patterns, and abnormal sensory processing (1). In addition to these typical features, motor dysfunction is increasingly being seen as one of the core features of autism (2, 3). Children with ASD often exhibit certain motor abnormalities in walking patterns (4), hand movements such as reaching and grasping (5), and eye-hand coordination (6), and their motor development is also much delayed (7). These motor deficits interfere with normal functional activities, which inevitably affect social interactions (8). For this reason, considering the need for a more reliable diagnostic process for ASD children, motor features could represent an effective precursor. Recently, several studies focused on using machine learning systems to identify autism motor patterns (9). For example, in the latest study, a deep learning latent variable model was proposed to identify children with ASD through motor abnormalities. This model could automate ASD detection and provide a new quantitative method to assess ASD (10).

Recently, research around autism has made great strides over the past decade in moving away from a medical deficit model to a neuro-divergent model that accounts for processing differences in the autistic phenotype. Within the neurodiversity movement, autism is conceptualized using the social model of disability which regards disability as resulting from a poor fit between the (physical, cognitive or emotional) characteristics of a given individual and the characteristics of their social context. A person is disabled not by their impairment, but by the failure of their environment to accommodate their needs (11).

Therefore, within the frame of a neuro-divergent model, to better improve motor deficits in children with ASD to some extent, motor skill acquisition is a good approach to help ASD children participate in physical activities and interact with coaches and other children. And this approach will create a much more abundant and interesting environment to them. Given the propensity for neuroplastic change in the nervous system, children with ASD may also benefit from this approach to improve functional performance (12).

However, previous studies have found that children with ASD have more difficulty in learning new motor skills than their peers (3), and some studies have suggested that this lack of competence in motor skill acquisition is itself a core symptom of the motor deficits seen in children with ASD (13). The negative effects arising from the difficulty in learning motor skills emphasize the need to create practical strategies to improve motor learning and performance in these children.

During the acquisition of functional motor skills, two types of learning processes occur simultaneously and are interdependent: explicit learning and implicit learning. In explicit learning, learners have a clear awareness of the motor knowledge being assimilated, while in implicit learning, the acquisition of information is not accompanied by a conscious awareness of the learned knowledge (14). Previous studies have shown that the implicit motor learning abilities of children with ASD are intact despite their deficient motor learning abilities. For example, a study compared the motor learning abilities of children with ASD on a serial reaction time task with typically developing children. The task distinguished between explicit and implicit processes of motor learning by participants being aware and not being aware of the sequence, respectively. Results revealed that children with ASD were able to acquire motor sequences implicitly rather than explicitly (15). These findings can be explained by the neural mechanisms involved in both types of motor learning. The right hemisphere is more dominant in implicit learning compared to the left, and there is an overlap between the dysfunctional areas of the left hemisphere and the explicit learning areas in children with ASD (16). Therefore, the ability to learn implicit motor sequences is preserved in children with ASD to a greater extent than the ability to learn explicit motor sequences.

In summary, children with ASD have specific motor deficits and have greater difficulty learning new motor skills, but their ability to learn implicit motor skills remains relatively intact compared to explicit motor skill learning. Therefore, from a practical teaching perspective, we can utilize implicit instructions to guide children with ASD in mastering new motor skills. In the following sections, we will briefly introduce several common implicit learning approaches in children’s motor skill learning and then summarize the current research on implicit motor learning in children with ASD. On this basis, we will propose future research perspectives to support the development of more effective approaches to teaching motor skills to children with ASD.

## 2 An overview of implicit motor learning methods in children

Commonly adopted implicit methods in previous studies of children’s motor learning include dual-task learning, errorless learning, the external focus of attention, and analogy learning (17).

The dual-task approach is the first attempt to cause implicit motor learning (14). In this paradigm, participants practice a primary motor task while performing a secondary task. The secondary task takes up a certain amount of working memory load and therefore prevents the development of declarative knowledge of the primary motor task. Dual-task participants typically learn the task and accumulate less knowledge compared to participants who do not perform the secondary task, suggesting that the task is acquired implicitly. However, the secondary tasks are relatively difficult and their use in practice has the potential to confuse learners. To address this issue, alternative learning paradigms have subsequently been developed that reduce the acquisition of declarative knowledge in motor learning and enable better application in practical contexts (18).

Errorless learning is a method aiming to minimize errors in the learning process. It is based on the theory that when an error occurs, the participant actively generates hypotheses on how to improve motor performance and tests the hypothesis in the next attempt. As such, knowledge is gained explicitly through this process (19). In errorless learning, limiting the environment to minimize performance errors leads to a limited need for hypothesis testing, which in turn reduces the involvement of working memory and the development of explicit knowledge. This approach could encourage movement exploration.

The third approach is the external focus (EF) of attention strategy, which refers to the learner’s attention being shifted to the effect or outcome of the action on the environment, rather than to the physical action as in the case of the internal focus (IF) of attention (20). Previous studies have demonstrated that practicing with an EF improves motor learning and performance (21). Moreover, meta-analysis has shown that an EF is more advantageous than an IF regardless of the stage of sports performance and learning test used, as well as the age, health status, and skill professional level of the participants (22). Theoretically, EF facilitates the process of automatic control, while IF is thought to interfere with automatic control.

The fourth approach is analogy learning, which involves integrating the complex structure of the skill into a simple biomechanical metaphor (23). This metaphor relies less on the manipulation of explicit verbal information and facilitates implicit learning of motor skills. Previous research has found that motor performance during the practice phase of analogy learning does not differ significantly from that of explicit learning. Thus, analogy learning overcomes the difficulty that motor performance arising from the other implicit methods is poorer than that of explicit learning. It also retains the advantage of providing stability in the motor performance arising from implicit learning, providing a more practical approach to motor skill learning. And in some case, if the instruction of analogy is about external focus, it can also belong to EF approach. This indicates that these implicit motor learning approaches are not complete independent, and they just emphasize different guiding perspective.

Implicit learning approaches have been shown to be effective and, in some cases, even more effective than explicit learning approaches in improving motor skill performance in adults (24). They are also suitable for children because they require fewer cognitive resources. In the following section, we will highlight recent research on the acquisition of motor skills using implicit methods in children with ASD.

## 3 Current approaches to implicit motor learning in children with autism spectrum disorder

Relatively few studies in the existing literature have directly applied implicit learning methods to motor skill learning in children with ASD. Thus, the current mini-review is a literature review rather than a systematic review. And the literature search was conducted using the following databases: PubMed, PsychINFO, EBSCO and Web of Science. The initial search was performed in June 2023 and updated in December 2023 using the following keywords: TS = (autism children OR ASD children) AND TS = (implicit OR analogy) AND TS = (motor learning OR action learning OR motor skill OR motor performance). We finally screened out five articles about implicit motor learning in ASD children based on these databases and Google Scholar (**Table 1**). The two main methods used are EF of attention and analogy learning.

**Table 1:** Studies on implicit motor learning in children with ASD.

Author (year)	Implicit method	Age range (years)	Task	Condition and sample size	Primary findings
Samsudin & Low (2017) (25)	EF of attention	7–10	Throwing boules	EF (n=5) IF (n=5)	EF group showed greater improvement by producing a closer distance between the boules and the jack compared to the IF group.
Tse (2019) (26)	EF of attention	9–12	Throwing beanbags	EF (n=22) IF (n=22) Control (n=21)	IF group showed better throwing performance than the EF group or the control group.
Asadi et al. (2022) (27)	EF of attention (during observational learning)	7–10	Throwing tennis ball	EF (n=12) IF (n=12)	EF group threw more accurately and performed better in the post-test than IF group.
Tse & Masters (2019) (28)	Analogy learning	9–12	Basketball shooting	Visual Analogy (n=12) Verbal analogy (n=12) Explicit instructions (n=12) Control (n=12)	Visual analogy group displayed more robust motor performance during transfer and retention than the verbal analogy, explicit instructions group, and control group.
Kok et al. (2021) (29)	Analogy learning and EF of attention (with instructions and feedback)	9–13 Students with special needs (including ASD)	Walking a slackline (balancing task)	Total n=115 Implicit group (analogies and EF) Explicit group (Explicit instructions and IF)	The explicit and implicit groups showed similar improvements overall. Verbal working memory was found to influence the effect of instruction and feedback methods on learning outcomes.

EF, external focus; IF, internal focus.

### 3.1 External focus of attention

As mentioned above, previous research has found that using an EF of attention facilitates skill learning and improves motor performance compared to an IF of attention in adults and children in general. However, few studies compare the effects of different attentional focus on motor learning in groups of children with abnormal development. Furthermore, recent findings relating to the attentional focus approach in children with ASD are inconsistent.

The study by Samsudin and Low (25) applied an external attention focus to instructions for a motor learning task in children with ASD. Ten children learned a modified-petanque game and were randomly divided into an EF or IF group. Petanque is a sport that requires manipulative skill (throwing) and visual-object control. The EF group was instructed to throw the boules in a “rainbow” trajectory, while the IF group was instructed to concentrate on the mechanics of the throwing arm. The target of both groups was to throw the boule and aim to land closest to the target for points. The results showed that the children in the EF group threw the boules more accurately in the post-test compared to the children in the IF group. The results support previous findings in the general population or other special groups of children. However, the main limitation of the study was the relatively limited number of participants. In Tse’s (26) study, the effect of using an EF or IF of attention was examined in children with ASD who were asked to complete a beanbag-throwing challenge. Compared to the previous study, this study had a better methodological quality. In study design, this study added a control group and there was a substantial increase in the number of participants, with 65 participants recruited in total and more than 20 participants in each group (EF, IF, and control). And this study provided more demographic statistics of participants and more detailed description of statistical methods. Children in the EF group were instructed to concentrate on the beanbag flight path, and the IF group was instructed to concentrate on the movement of their throwing arm, while the control group received no attentional focus instructions. The results showed that the throwing performance of the IF group was significantly better than that of the EF group or the control group in the retention and transfer tests. This finding contrasts with what is usually observed in typically developing (TD) children and children with intellectual disabilities (ID). Children with ASD may rely more on proprioceptive sensations than on vision to direct movements in accordance with the IF instructions, while the EF instructions may have focused attention on the movement effect and required children to modify their movements in the retention and transfer tests by using their vision more. To further examine this hypothesis, future research should investigate the connection between attentional focus and sensory feedback in both TD and ASD children.

Observational learning is a common manipulation in the motor instructions used in children with ASD, and video demonstrations are more effective in teaching children with ASD than live demonstrations (30). In a study on tennis ball throwing in ASD children, the researchers effectively combined attentional focus instructions with an observational learning approach (27). Here, participants were assigned to an EF or IF group, and both were taught using a video demonstration with appropriate instructional language relating to the study condition. Detailed apparatus, task, and demographic information for children in EF and IF groups were provided in this study. Children in the EF group threw more accurately and performed better in the post-test. However, the results of this study were inconsistent with those of Tse (26). The authors proposed that since children with ASD are socially impaired, it may have been uncomfortable for them to focus on the body movements of the demonstrator in the video. Therefore, children with ASD are more likely to benefit from teaching with an EF of attention rather than IF when watching video demonstrations.

Although the motor tasks in these studies were administered in lab rather than outdoor sports environment, they still required some fundamental motor skills just like throwing, which had a higher ecological validity than other tasks like SRTT. However, we think that they only focused on simple throwing tasks, and more research is needed to better understand how instructions with different attentional focus affect children with ASD in performing different and more complex motor tasks to obtain richer and clearer findings.

### 3.2 Analogy learning

Numerous studies have shown that motor learning is strongly related to working memory (31), and the chunking feature in analogy learning can help learners release some working memory resources, allowing them to handle the additional cognitive demands imposed by secondary tasks, thus producing better motor performance in multitasking conditions (32). Given the benefits of analogy learning found in previous studies of motor learning in TD children and that children with ASD generally have a smaller working memory capacity (33), there have been attempts to use analogy learning to help children with ASD acquire motor skills. In the study by Tse and Masters (28) on the learning of basketball shooting skills, 48 children with ASD were randomly divided into a visual analogy group, a verbal analogy group, an explicit instructions group, and a control group. A portable hoop and a regular size 5 basketball weighing 25% less than a standard basketball were used to suit the motor ability of children with ASD. All groups, except the control group, received instructions about a basketball shooting task during the learning phase. This study also had a detailed description of participants and statistical methods. It was found that the performance on the retention and transfer tests decreased in the verbal analogy, explicit group, and control group compared to their performance at the end of the learning phase, while in comparison, the performance in the visual analogy group did not decrease. These findings suggest that visual analogy may be an effective teaching method in helping children with ASD learn motor skills. In addition to demonstrating the effectiveness of analogy learning in this context, the findings suggest that analogy learning in the visual modality is more directly helpful to children with ASD than analogy learning in the verbal modality. Additionally, children with ASD have a limited verbal working memory, and thus, verbal analogies may not be sufficient to induce the motor learning benefits demonstrated in TD children (34).

Although many previous studies have implemented the two approaches of analogy learning and EF of attention separately, the cognitive mechanisms of the two are intertwined. When using implicit motor learning instructions, it is possible to use a combination of the two approaches. For example, in a study on physical education for children with special needs, the researchers recruited a group of primary school students with learning difficulties and behavioral or social disorders, including children with ASD, attention deficit hyperactivity disorder, and reactive attachment disorder. Because this study did not provide detailed information on children with ASD, we could not make conclusion about analogy learning for ASD children directly from this study. However, we can get some enlightenment about combination of two approaches of analogy learning and EF of attention in the future studies. In this study, participants completed a balancing task (29) having been randomly divided into two groups, with the explicit group receiving specific and detailed explicit instructions and feedback, as well as instructions on the IF of attention, and the implicit group receiving analogy instructions and feedback, as well as instructions on the EF of attention. The to-be-learned task was to walk a slackline (length: 390 cm; width 35 mm; height: 31 cm), using as little support as possible. The slackline was stretched as tight as possible on a slackrack with help of a tension rattle. The moderating role of working memory capacity (WMC) was also explored. The explicit and implicit groups showed similar performance improvements overall. However, the study also found that for the explicit group, larger verbal WMC was associated with greater improvement in balance outcomes, whereas for the implicit group, verbal WMC was negatively associated with improvement in balance outcomes. In some participants, verbal analogy instructions may not promote motor execution due to comprehension problems. The results are similar to those of Tse and Masters (28), which suggested that when teaching motor skills to children with special educational needs, the applicability of different types of analogies should be considered when using instructions, as well as the effect of individual abilities such as WMC on the effectiveness of explicit and implicit teaching methods.

## 4 Future directions

In summary, the application of implicit learning to the acquisition of motor skills in children with ASD is still an emerging field. However, the results of some recent empirical studies have shown that the implicit learning approach can improve motor skill learning in children with ASD. To better apply this approach to the practical teaching of motor skills in children with ASD, we need to refine the existing methodologies. And we provide our summary of proposed future research directions and consideration of the implications for real-world teaching in the final section.

First, we should enrich the selection of task types and refine the measurement indicators of sports performance in the future. Most studies on implicit motor learning in children with ASD have used goal-based tasks, such as throwing tasks, and only one study used a whole-body balance task. In addition, existing studies have used only a single measure of motor performance, which were mainly outcome-oriented measures and lacked process-oriented measures, which is a key factor in evaluating fundamental movement skills in children. Especially for children with ASD, who have much more difficulties in learning new motor skills and need more process-oriented measures to reflect their learning process. Therefore, we should consider the process-oriented measurement and incorporate more indicators such as the effectiveness of sports performance, sports efficiency, and the degree of automation of motion control. For example, we can assess the effectiveness of sports performance via objective performance accuracy and a subjective evaluation of sports performance quality could be provided by experts. We can measure sports efficiency by physiological indicators such as oxygen consumption, muscle activity, and contraction, and the degree of automation of sports control could be measured using a dual-task paradigm (22).

Second, it is necessary to ensure the effectiveness of the guidance, that is, to ensure that ASD children really do learn effectively when using implicit methods. Some existing studies did not complete a manipulation check on the guidance provided or simply relied on subjective report from the participants. In future research, we can use objective physiological indicators alongside subjective reports. For example, when an external focus of attention strategy is used, an eye tracker could be used to conduct a manipulation check on the focus of attention, and to further understand the change in attention focus as required by the instructions given.

Third, ASD children’s individual characteristics should be considered. Most of the previous studies were carried out at the group level, and individual differences were not fully taken into account, including age, conscious control propensity, and WMC. Tse and van Ginneken (35)showed that children with a high propensity for conscious control performed better in an IF group, while children with a low propensity for conscious control performed better in an EF group, suggesting that children’s motor skill acquisition is most effective when the mode of instructions provided is consistent with their propensity for conscious control. Therefore, in future studies, we need to evaluate the effectiveness of the implicit teaching method in light of the individual characteristics of each ASD child.

Finally, to improve ecological validity, we should increase the use of real-world physical education teaching scenarios, and also organically combine several methods of implicit learning to reflect the mixed teaching methods used in the real world and to maximize the advantages of implicit learning. For example, teachers could use analogies that point to an EF of attention, or they could provide learners with external focus guidance after an errorless initial practice (18).

## 5 Conclusion

In conclusion, children with ASD show impairments in motor skill learning compared to TD children. However, they retain a greater ability to learn implicit motor skills than explicit motor skills. There is a need to find more efficient approaches to improve motor functioning in children with ASD to facilitate their participation in physical activity and social interaction. In the current literature review, we summarized the small number of empirical studies in this field and analyzed the effect of the two main implicit methods (EF of attention and analogy learning) used to date in children with ASD. Although some of the results were inconsistent, the overall effects provide inspiration for working to adopt these implicit motor learning skills for the benefit of ASD children. Future studies should aim to provide a solid foundation for the effective use of these implicit methods in various real-world situations for children with ASD.

## Author contributions

The author confirms being the sole contributor of this work and has approved it for publication.
